# Use of phase angle as an indicator of overtraining in sport and physical training

**DOI:** 10.1186/s12967-024-05918-w

**Published:** 2024-11-29

**Authors:** Giuseppe Annunziata, Antonio Paoli, Evelyn Frias-Toral, Stellario Marra, Francesco Campa, Ludovica Verde, Annamaria Colao, Henry Lukaski, Daniel Simancas-Racines, Giovanna Muscogiuri, Luigi Barrea

**Affiliations:** 1 Facoltà di Scienze Umane, della Formazione e dello Sport, Università Telematica Pegaso, Via Porzio, Centro Direzionale, Isola F2, Naples, 80143 Italy; 2 Department of Biomedical Sciences, University of Padua, Padua, Italy; 3 School of Medicine, Universidad Espíritu Santo, Samborondón, 0901952 Ecuador; 4 Department of Public Health, University of Naples Federico II, Via Sergio Pansini 5, Naples, 80131 Italy; 5 Unità di Endocrinologia, Diabetologia e Andrologia, Dipartimento di Medicina Clinica e Chirurgia, Università degli Studi di Napoli Federico II, Via Sergio Pansini 5, Naples, 80131 Italy; 6 Diabetologia e Andrologia, Dipartimento di Medicina Clinica e Chirurgia, Centro Italiano per la cura e il Benessere del Paziente con Obesità (C.I.B.O), Unità di Endocrinologia, Università degli Studi di Napoli Federico II, Via Sergio Pansini 5, Naples, 80131 Italy; 7 Cattedra Unesco “Educazione Alla Salute E Allo Sviluppo Sostenibile”, University Federico II, Naples, 80131 Italy; 8 Department of Kinesiology and Public Health Education, Hyslop Sports Center, University of North Dakota Grand Forks, Grand Forks, ND 58202 USA; 9 Centro de Investigación en Salud Pública y Epidemiología Clínica (CISPEC), Facultad de Ciencias de la Salud Eugenio Espejo, Universidad UTE, Quito, 170129 Ecuador; 10 Department of Wellbeing, Nutrition and Sport, Università Telematica Pegaso, Centro Direzionale Isola F2, Via Porzio, Naples, 80143 Italy

**Keywords:** BIA, BIVA, Overreaching, Fatigue, Inflammation, Overtraining, Acute and chronic fatigue

## Abstract

The use of bioelectrical impedance analysis (BIA) is now well established in healthcare as an essential support tool for patient management in various clinical settings. Its use in sports is rapidly expanding due to the valuable insights it offers, helping to better structure athletes’ diets and training programs, thereby optimizing their performance. In the context of sport, however, there is a consensus regarding the importance of proper interpretation of BIA-derived data, which cannot be limited to mere estimation of body composition. In this sense, therefore, the evaluation and interpretation of raw bioelectrical parameters, including resistance, reactance, and phase angle (PhA) is of relevant importance. The assessment of PhA is particularly significant in the context of sports, as it is closely linked to key factors such as muscle mass, strength, and overall muscle quality. However, the existing relationship between PhA and systemic, and loco-regional inflammation, which, in a broader sense, is the rationale behind its use for assessing and monitoring localised muscle damage. Thus, the importance of PhA monitoring during training becomes evident, as it plays a crucial role in assessing and potentially identifying functional impairments, such as overtraining syndrome, as well as muscle injury and related changes in fluid distribution, at an early stage. The aim of this review is to provide the scientific basis necessary to consider the use of whole-body PhA as an indicator of overtraining.

**Supplementary Information:**

The online version contains supplementary material available at 10.1186/s12967-024-05918-w.

## Background

The use of bioelectrical impedance analysis (BIA) in clinical practice for the estimation of hydration status, nutritional status (*via* bioelectrical impedance vector analysis, BIVA), and prognosis is now well established, given its advantages in terms of rapidity of execution of the method, processing and interpretation of the information obtained, but also for its non-invasiveness and low cost [1, 2]. The BIA method measures the impedance (Z) to the flow of an alternating current, which is directly related to body’s fluid content and its distribution among intra and extracellular spaces [1]. A low amplitude, alternating electric current flows through cables connected to electrodes or conductive surfaces placed on the skin. A phase-sensitive device usually operating at 50 kHz measures resistance (R) and reactance (Xc), which are then used to calculate Z and phase angle (PhA) [3]. These measured parameters provide information on the state of hydration (in terms of total body water [TBW], intracellular water [ICW], and extracellular water [ECW]), based on the assumption that the R opposed to an electric current is inversely proportional to the content of TBW and electrolytes [1, 2]. The use of predictive formulas (multiple regression models) that take into account both the information just described and certain anthropometric (e.g. body weight and height) and demographic parameters (e.g. age, gender, and ethnicity) of the subject will make it possible to estimate body composition (BC) [1, 3, 4].

Evidence emphasises the importance of monitoring changes in bioelectric parameters detected by BIA, especially in specific clinical settings, which disregards the mere estimation of BC [5, 6]. This approach is particularly important when it is considered, first, that often the formulas used to estimate BC may be inaccurate for subject-specific reasons, as, for example, in the case of subjects with morbid obesity, in which a major expansion of body fluids is observed, as well as in certain pathological conditions that are characterized by important states of dehydration [7–10]. By thoroughly understanding the biological significance of the raw BIA parameters (R, Xc, Z, and PhA), thus, from their assessment and, above all, monitoring over time, it is possible to obtain important information regarding specific indicators of health status that are independent of BC.

Beyond the clinic, sport also represents an important field of study in which application of BIA is increasingly prioritized [1]. Estimation of BC with BIA in sport has been surpassed by the recognition of the importance of monitoring variations in the athlete’s bioelectrical characteristics as related to training and performance, notably PhA [11]. Reference percentiles for PhA are available for various sports, providing valuable benchmarks for performance and health monitoring [12]. Indeed, as reported in a systematic review, PhA values vary significantly between athletes and non-athletes, being markedly higher in the former than in the latter, likely because of greater body cell mass (BCM) and consequently muscle mass. Similarly, within the same sport, PhA values are higher in athletes with a better level of performance suggesting, therefore, the usefulness of measuring this parameter in order to assess muscle quality and function allowing discrimination between well-trained and untrained subjects [11]. These observations, therefore, allow us to translate the concept of PhA into a broader context of exercise interpretation, allowing us to posit or hypothesize its role as a true marker of “training quality.”

We describe “training quality” as a comprehensive assessment of activity, which includes the three fundamental components of physical training: intensity, duration, and frequency. An imbalance in one or more of these variables can result in an excessive (and unhealthy) increase in training load, which, in some extreme cases, can border on the development of overtraining syndrome (OTS), whose negative repercussions on psycho-physical health status are now well known [13]. Hence, we posed the question: *can PhA play a role in early identification and monitoring of acute fatigue (overreaching)?*

To answer this question and substantiate this concept empirically observed in routine clinical practice, the available literature was critically evaluated for a narrative overview outlining the state of the art with regard to the hypothesis of using PhA variations as indicators of overtraining (OT) in sport and physical training. To our knowledge, although no studies measuring directly PhA values under OTS conditions have been conducted, accumulating evidence is available regarding the physical, biological, and physiological principles that associate PhA to this detrimental condition for the athlete. In particular, systematic reviews, and meta-analyses have been published on key topics such as the use of BIA in sports, the use of PhA as an indicator of muscle characteristics or as a marker of inflammatory status [14–18]. We utilized this information for the theoretical basis of our hypothesis to justify the use of PhA as an indicator of potential OT, and a focus on a particular type of training, the resistance training resistance (RT), was carried out, given its clearly reported effects on muscle hypertrophy and strength, as well as on PhA changes [14, 19]. Although many studies on OT refer to aerobic or endurance training, resistance exercises represent an important component that can result in OT [13, 20, 21]. A meta-analysis that evaluated the association between PhA and physical activity levels (PAL) showed that RT represents the type of exercise evaluated in most of the studies considered [22]. Furthermore, it appears that only one study evaluated the effect of changes in training volume and intensity, noting that RT results in improved PhA [23], but leaving the question related to the best dose-benefit ratio unresolved [22].

### Beyond body composition estimation: the use of BIA raw parameters in the context of sport

#### The physics of BIA

The BIA method is based on Ohm’s law. The potential difference (or voltage drop) across a conductor is directly proportional to the resistance the conductor opposes to the flow of current. The relationship between resistance, voltage and current intensity is given by the equation R = V/I, where R represents resistance (Ohms), V the voltage (Volts) and I the current intensity (Amperes) [2, 24]. Ohm’s law also states that the R of a cylindrical conductor is directly proportional to its length (L) and inversely proportional to the cross-sectional area (A). This means that as the L of the conductor increases, the R will increase, while as the A increases, the R will decrease. This relationship can be expressed as R∝L/A. By introducing a proportionality constant (ρ), it can obtain the Ohm’s second law, which can be written as R = ρ L/A. This law relates the R, L and A of a conductor. Ohm’s law is fundamental to the BIA technique, as it allows BC to be estimated by measuring the body’s electrical R and L. In practice, it is easier to measure the height of a subject than the length of the conductor, so body volume can be estimated using the ratio H^2/R, where H represents height. This empirical relationship links fat-free mass (FFM) to the ratio H^2/R [7]. It must be emphasised, however, that the human body cannot be regarded as a homogenous cylinder with constant conductivity, so it is essential to take into account specific coefficients that consider the actual geometry, thus, depending on factors such as the anatomy of the body segment under consideration [7].

#### BIA raw parameters

Although BIA is commonly used in clinical practice to estimate BC, it is important to emphasize that accurate results can only be achieved when equations developed using multi-component models are applied [25]. This approach helps avoid potential errors related to hydration assumptions, as hydration levels are not always consistent both within and between populations.

This suggests that the estimation of BC is less important than the identification of biomarkers of BC changes associated with cellular function and health status. Raw bioelectrical measurement values can also provide meaningful information parameter without the need for further empirical transformation [8].

Some BIA devices can directly measure bioelectrical parameters such as R and Xc, which correspond to the resistive and capacitive properties of tissues, respectively. R indicates how much a conductor opposes the flow of current, making it inversely proportional to TBW. Therefore, a higher amount of TBW and electrolytes in the muscles facilitates the conduction of the applied alternative current, and fluctuations in TBW significantly impact the measured value of R. The Xc component, on the other hand, reflects the delay or hindrance in current penetration of cell membranes and interfaces of tissues [1].

From R and Xc, another important parameter emerges: PhA, defined by the formula PhA = arctan(R/Xc) × (180°/π) [26], where, in the context of sports, R and Xc are commonly measured at 50 kHz frequency [16]. Using a phase-sensitive BIA instrument, PhA is a direct measure of the ratio of Xc to R, providing insight into the quantity and functionality of cell membranes, reflecting BCM as well as fluid distribution [11].

PhA parameter depends on several factors [27], such as cell content, body fluids, and the integrity and permeability of cell membranes [16]. In other words, the PhA, by providing information about (i) intra- and extracellular fluid distribution [10, 28, 29] and (ii) membrane integrity and cell size [27, 30], makes it possible to identify the possible presence of cell damage.

Given the previous premises, therefore, it is clear that the evaluation of the raw BIA parameters (R, Xc and PhA) can be particularly useful in specific settings, such as the sport contexts to identify and monitor both acute and chronic effects of physical training. After intense sport activities, in fact, hydration status can be altered, making accurate assessment of TBW with BIA difficult [31]. This can lead to inaccurate estimates of BC. In such situations, the use of raw BIA parameters is recommended to quickly monitor changes in TBW, offering a viable alternative to indirectly calculated parameters [32–35]. In this sense, studies by Mala and colleagues showed a significant reduction in Xc and PhA in judo athletes after rapid weight loss due to dehydration [36]. It is possible to speculate that this reduction in PhA indicates decreased cellular integrity, worse membrane condition and altered fluid distribution [35].

Although, therefore, the importance of assessing the raw BIA parameters (R, Xc, and PhA) in sport has been clarified, in the following paragraphs, and as the focus of this article, we will dwell on the biological significance of PhA, understood as, in a broader sense, a ‘surrogate’ or ‘cumulative indicator’ of changes in R and Xc.

The evidence below refers to whole-body BIA measurements, thus to assessments of total body PhA values. It is worth emphasising, however, the importance of evaluating and interpreting regional raw parameters as well, which can be obtained by means of localised phase-sensitive BIA measurements (L-BIA), capable of providing information on muscle quality and fluid distribution at the level of individual body locations or regions [18]. Following specific protocols for electrode placement and timing of the analysis, in fact, through L-BIA the observation of R, Xc and PhA variations allow the identification of tissue (muscle) damage and regional fluid disturbances. In particular, in the case of a muscle injury, reductions in (i) R, (ii) Xc and PhA are observed with respect to the contralateral body district, reflecting, respectively, the localised accumulation of fluid (post-traumatic oedema) and the presence of tissue/muscle damage as a consequence of the cell membrane disruption [18]. These changes in bioelectric parameters were confirmed by both in in vitro experimental observations and human comparisons with diagnosis imaging techniques [18, 37]. L-BIA, therefore, represents a valuable new approach for the early identification of muscle injuries and staging of severity [18]. At the same time, however, the monitoring of the variations of the raw parameters at a locoregional level (e.g. at the level of the limbs or muscular districts most involved in the athletic gesture in the various sports disciplines) may also represent a valid tool for the identification of a sort of ‘localised OR/OT’ or, at least a ‘muscular suffering’, therefore, referring to a high stress on a single district that could, if perpetuated, more easily predispose to injuries. The use of L-BIA, therefore, could acquire a preventive, as well as evaluative, connotation.

Confirming the importance of evaluating BIA raw parameters as a non-invasive tool to identify and assess exercise-induced tissue damage, a recent study (although not conducted with the L-BIA technique) showed that R, Xc and PhA were lower in the limb involved in exercise (eccentric exercise under experimental conditions); moreover, Xc values correlated negatively with urinary titin N-terminal fragment levels [38].

An overview on the biological significance of BIA raw parameter is reported in Table 1.

**Table 1:** Biological significance of BIA raw parameters. Abbreviations: Bioelectrical impedance analysis, BIA

BIA raw parameter	Biological significance
Resistance, R (Ohm)	It reflects the resistance opposed by a biological conductor (tissues) to an alternating current. It is inversely proportional to total body water. In L-BIA, its reduction (compared to the contralateral body district) indicates localised accumulation of fluid (localised oedema)
Reactance, Xc (Ohm)	It reflects the electric properties deriving from the capacitance of cell membranes. In L-BIA, its reduction (with concomitant reduction in PhA) reflects the presence of tissue damage as consequence of cell membrane disruption.
Phase angle, PhA (°)	It refers to the angle of impedance to an alternating current. It reflects the ability of current to flow across the cell membranes, and the intra-/extracellular ion distribution. It provides information about quantity and functionality of cell membranes, reflecting body cell mass. Its reduction suggests decreased cell integrity, worse membrane condition, and altered fluid distribution, and is associated with increased inflammation.

#### PhA for the evaluation of physical activity and fitness

Observational studies reveal that PhA is a novel indicator of physical activity, training and fitness [22, 39, 40]. The biological basis for this relationship should be found in the effects of exercise on (i) improved cell membrane integrity and function, (ii) changes in intracellular composition, and (iii) increased tissue capacity [27, 41]. As described above, PhA is an indicator of cell health, membrane integrity and cell function, thus it finely describes the effects of exercise on cell health and, consequently, on global whole-body health [22].

Another mechanism to explain this relationship lies in exercise-induced morphofunctional changes at the cellular level. We refer, essentially, to an increase in BCM and muscle strength [15, 22, 42]. An increase in BCM implies an increase in metabolically active cell mass [42], that is, directly involved in the processes of oxygen consumption, carbon dioxide production, and energy expenditure [39, 40]. The increase in PhA, therefore, is understandable if its significance as, precisely, an indicator of BCM is taken into account [43, 44]. On the other hand, the increase in muscle mass describes an increase in tissues that, physiologically, are characterized by an abundance of water and electrolytes and that, therefore, represent excellent conductors; this, from a bioimpedance point of view, is reflected in a reduction in R [8, 45] and an increase in Xc values [7, 45], resulting in a mathematical increase in PhA [46].

The ability to perform exercise is defined by health-related fitness (HRF), a theoretical construct that summarises several key components, such as cardiorespiratory and musculoskeletal fitness, flexibility, balance and speed [47]. In this contest, therefore, the mechanisms considered to describe the relationship between PhA and exercise allow us to think of this BIA parameter also as an indicator of fitness. Supporting this theoretical concept is a systematic review demonstrating the direct association between PhA and aerobic fitness in subjects of various age groups, gender and health status [15]. Similarly, a recent study demonstrates that PhA is able to predict all measures of HRF, resulting in a valid assessment of musculoskeletal fitness, which was higher in subjects falling in the highest tertile of PhA [48]. These results, therefore, suggest the importance of assessing PhA also for monitoring the effects of exercise, in all subject classes.

It is important to emphasise, however, that these concepts should always be interpreted from both a qualitative and a quantitative point of view. Although, as mentioned above, physical exercise in itself brings about changes at the cellular level that, on the whole, are reflected in a qualitative improvement of the tissues and, by translation, of the overall health, from a quantitative point of view it is possible to consider a bimodal action: an ‘inter-individual on-off’ effect describing the observable differences between those who exercise regularly and those who do not, and an ‘intra-individual’ effect dictated by the variations observable during exercise, in acute and chronic conditions. This dual meaning of exercise can be monitored by PhA. With regard to the first effect, in fact, some authors support the usefulness of PhA as an indicator of PAL [49]. With regard to the second point, on the other hand, the conclusions are largely speculative, and supported by evidence of an initial negative effect of exercise on cellular health [50], followed by a phase of long-term individual adaptation [51] to compensate for this effect in the acute, resulting in improved cellular health [52]. Interestingly, Nabuco et al. [53] demonstrated that soccer players with lower PhA were those who exhibited higher fatigue levels during sport-specific tests, regardless of other body composition characteristics. Supporting this, Reis et al. [54] demonstrated that during a training macrocycle in swimmers, reductions in PhA were recorded when training load was high, accompanied by decreases in performance. Subsequently, when the training load was reduced, PhA increased beyond baseline levels, along with improvements in performance, measured by faster 50-meter swim times. Similar patterns of change have also been reported in a longitudinal study on soccer players [55], where a decrease in PhA was observed after the preparatory phase, followed by a subsequent increase during the mid-season competition phase. Finally, the end of the season was marked by a further decline in PhA. What has been reported, therefore, would suggest an initial reduction in PhA values during exercise (in acute), followed by their increase (in chronic). The theoretical and mechanistic basis of this possible speculation, however, will be described in detail in the following sections.

#### Variations of PhA values during the resistance training

The increase in muscle fibres due to training-induced hypertrophy decreases R values, as muscle mass is an excellent conductor [42]. Based on this observation, certain activities such as RT, a recognised method for promoting muscle growth and remodelling [56], can improve PhA values. To better understand this relationship, it is necessary to refer to a functional response of the cellular structure to RT, which describes a kind of variability in fluid distribution [14], represented by an initial decrease in ICW (in the immediate post-training phases) [57], followed by a subsequent increase (in the recovery phases) [58]. This phenomenon is mostly related to an increase in intramuscular glycogen stores, which binds water in a 1:3 ratio (grams/grams) [14, 59]. Prolonged RT sessions thus lead not only to acute muscle changes, but also to long-term intracellular adaptations [14].

Pioneering studies in this regard showed that PhA values were significantly higher in bodybuilders than in their counterparts who were not regularly trained with RT [60]. Subsequent observations showed that it was RT aimed at hypertrophy, per se, that led to significant increases in PhA, both in elderly women [61] and in young men and women, regardless of the participants’ gender [62]. According to the authors of these studies, changes in PhA values should be considered in the context of a change in hydration status. Considering, in fact, PhA as a ratio between Xc and R, the increases observed in these studies can essentially be traced back to a reduction in R (in the denominator, in the PhA formula), as the values of Xc remained unchanged throughout the experimental periods [61, 62]. Thus, RT, by inducing an increase in muscle glycogen stores, leads to an improvement in cellular hydration (increase in ICW) [1, 63], which, in turn, justifies an increase in PhA values obtained by reducing its resistive component (R) [61, 62]. This increase in ICW seems to be justified by the particular sensitivity of fast-twitch muscle fibres to osmotic changes, a characteristic that is probably due to an increased presence of aquaporin-4 transporters [64]. On the other hand, it should be remembered that it is precisely the improvement in intracellular hydration that could play a key role in muscle hypertrophy, since it represents a stimulus for pathways that promote protein synthesis, as well as for those that slow down protein degradation [65, 66]; similarly, the increase in ICW could be a determining factor in stimulating the proliferation of satellite cells, facilitating their fusion with myofibrils during hypertrophy [67], thus justifying the hypertrophic effect of RT.

In addition to the RT-induced changes in intra/extracellular fluid distribution described above, a recent study provided interesting evidence for a more complete understanding of the biological significance of PhA, and which can be translated into various contexts, including sport. Huemer et al. [68] demonstrated that PhA is independently associated with a proteomic profile, in particular with protein markers of cell growth and muscle hypertrophy. These results, therefore, underpin the role of PhA in reflecting BCM [68]. In this context, lower BCM levels, together with altered selective cell permeability, result in lower PhA values, suggesting impaired muscle quality, in particular lower muscle strength [14]. Interestingly, RT is considered a type of exercise that promotes cellular adaptation, resulting in improved cell health [14]. This reinforces the role of RT in increasing PhA values as a consequence of qualitative and quantitative improvement in muscle mass and strength.

Another aspect to be taken into account with regard to the observation of an increase in PhA following RT concerns the change in the geometry of body districts and, consequently, tissues. Indeed, RT, by stimulating muscle hypertrophy, causes a change in the cross-sectional area of this tissue which, in turn, influences the biological current course [14], as demonstrated in a recent study in which it was observed that increases in the cross-sectional area of the thigh correlated positively with intracellular R-index, Xc and PhA following a 24-week RT programme [69].

### The potential role of PhA in overreaching/overtraining components

#### Overreaching and overtraining

It is well established that improvement in physical performance requires the athlete to engage in a progressive increase in training load. Additionally, the increase in workload must be interspersed with an adequate rest period to balance the training. Failure to include progressive training load with adequate rest periods can result in the conditions of overreaching (OR) and OT that may be identified with BIA measurements.

OR and OT are conditions related to increased training load and exposure to stressors not directly related to training. According to the European College of Sport Science and the American College of Sports Medicine, OR is a temporary reduction in performance, which may or may not be accompanied by physical and psychological signs, with recovery occurring in a few days or weeks. OT, on the other hand, represents a more lasting condition, with recovery taking weeks or months. The time factor is therefore central in the distinction between OR and OT [13].

OR can be considered a normal part of the physiological adaptation process [13], which, if it resolves in the short term (about two weeks) and leads to improved performance (supercompensation), is termed Functional Overreaching (F-OR). Conversely, if it continues for a longer period (3–4 weeks) without improvement, it is classified as Non-Functional Overreaching (nF-OR), representing a possible prelude to the development of OTS [70].

However, the definition of OR and OT cannot be reduced to the time factor alone, as the complexity of human physiology and the individuality of athletes make it difficult to make a clear distinction between the two conditions. Moreover, there are no specific diagnostic tests or biomarkers recognized as gold standards for identifying OR or OT, which further complicates the diagnosis, which is based on history, performance decrement, and mood disorders, to the exclusion of other causes [13].

Interestingly, however, the hormonal changes underlying the physical signs of OR and OT are also responsible for alterations in hydration status and fluid distribution [70–72]. In this context, therefore, a cross-sectional consideration of the physical consequences caused by OR and OT, and reflected in changes in the body’s bioelectrical parameters is plausible, making BIA potentially valuable in identifying OTS.

The identification of biomarkers for OTS, thus, is crucial to prevent and diagnose OTS, especially in endurance sports that require a high volume of training, such as swimming, triathlon, cycling and rowing, as well as short-term explosive or combat sports. As well, the definition of the nature of OTS and the identification of personalised signatures for OTS are urgent and essential field of research with significant implications for the clinical management of athletes [73].

#### The role played by oxidative stress and inflammation

In addition to decreased performance, mood changes and neuro-immuno-endocrinological alterations, variations in the inflammatory and oxidative states are another aspect closely linked to OTS, necessary for monitoring and early identification, and clearly identified by PhA changes [16].

Free radicals are reactive molecules naturally produced in the human body, capable of exerting both positive and negative effects [74]. Although the well-established beneficial effects of regular physical activity, there is evidence associating chronic excessive training with elevated levels of inflammatory markers and oxidative stress, leading to OTS when it is combined with inadequate recovery [73]. In this sense, Reactive Oxygen Species (ROS) play a critical role in initiating exercise-induced muscle damage and subsequent acute muscle inflammatory response [75].

Intense muscular work produces significant amounts of ROS. To prevent oxidative stress, the body uses endogenous antioxidant defences that either prevent ROS formation or neutralize free radicals [76]. An imbalance between free radical production and antioxidant defences leads to oxidative stress. Training, thus, can positively or negatively affect oxidative stress depending on training load, specificity, and baseline training levels. Additionally, oxidative stress is implicated in muscular fatigue and OT [74], and is associated with decreased physical performance, muscle damage, and OT [76].

Similarly, during the acute training response, peripheral cellular mechanisms involve associated cytokine and hormonal reactions. Glycogen deficiency is linked to increased local cytokine expression (interleukin-6, IL-6), decreased glucose transporter expression, increased cortisol, decreased insulin secretion, and β-adrenergic stimulation. Muscle damage and repair processes may involve the expression of inflammatory cytokines (e.g., tumor necrosis factor-α, TNF-α) and stress proteins (e.g., heat shock protein 72) [77, 78].

In this sense, single bouts of exercise increase oxidative challenge, while regular exercise decreases it. On the other hand, excessive exercise and OT lead to harmful oxidative stress, representing the extreme end of the hormetic response curve. Biological systems, indeed, respond to stressors in a U-shaped curve, with physical exercise also evoking this hormesis response. The two endpoints of the hormesis curve, inactivity and OT, result in decreased physiological function [79].

#### PhA as a surrogate biomarker of systemic inflammation and oxidative stress

As extensively described above, OR and OT conditions share an increase in inflammation and oxidative stress as a cause (or consequence) of the plethora of biological and physiological alterations underlying their characteristic symptoms. Inflammation and oxidative stress, in fact, are intimately interconnected, sharing specific signalling pathways and, together, can cause tissue damage and contribute to the development of several chronic diseases [16, 80]. In this context, inflammation acts as a homeostatic mechanism that is triggered in response to negative external (or internal) stimuli, such as damage, injury and infection, in order to restore a state of equilibrium. This response, triggered by the immune system, is therefore a defence mechanism, generally transient and mostly related to acute conditions. When, however, the negative stimulus continues over time, inflammation becomes chronic, causing tissue damage [16, 80, 81]. It seems clear, therefore, that both baseline assessment and monitoring of the inflammatory state over time provide valuable information on the subject’s state of health in different clinical settings, acquiring particular importance in sport as well. It must be emphasised, however, that this monitoring is carried out by means of the dosage of specific biomarkers of inflammation that require invasive (blood sampling) and costly (laboratory analysis) tests that may delay assessment, prognosis and possible treatment [16]. The use of surrogate markers that do not provide detailed information on the levels of these markers, but rather essential indications to monitor the inflammatory state by following its course over time, is essential. Amongst these, BIA has emerged as an alternative, low-cost method capable of providing real-time results regarding the progress of the subject’s inflammatory state [16, 82]. Plenty of evidence, in fact, has associated PhA with the inflammatory state, observing significant correlations between the values of this BIA parameter and the levels of markers of inflammation (including IL-6, TNFα, and CRP) in various clinical settings [27, 82–90], as well as following different interventions, such as, for example, ketogenic diet [91], recognised for its anti-inflammatory potential [92–94]. In the context of this extensive literature, the first study to assess the correlation between PhA and hsCRP values in a large number of adult subjects (over 1800 subjects) demonstrated the existence of an inverse relationship between these two parameters. Furthermore, the researchers calculated cut-offs for PhA values capable of predicting the presence of elevated hsCRP values that were found to be ≤ 5.5° in men and ≤ 5.4° in women [83]. Overall, experimental findings suggest the PhA as a valid indicator to monitor inflammatory processes in healthy individuals and clinical patients [41, 95].

To understand the rationale behind this relationship between PhA and the inflammatory state, it must be remembered that during inflammation and increased oxidative stress, the substances produced and released (e.g. interleukins and oxidising substances) cause a disruption of cell membranes by altering the hydro-electrolyte balance between intra- and extracellular spaces [80]. This imbalance that is created influences both the resistive component (associated with the state of hydration of the tissue) and the capacitive component (related to the capacity of the cell membranes) resulting in changes in PhA. In this context, therefore, PhA is an indicator of cell membrane integrity [27, 30] and, by translation, a surrogate indicator of inflammatory and oxidative abnormalities [16, 82]. More specifically, during inflammation there is, on the one hand, a decrease in Xc as a response to reduced membrane capacity and, on the other hand, a reduction in R as a result of an expansion of extracellular fluid. This results in a reduction of PhA as a ratio of Xc to R, due to a greater reduction of Xc than R. Hence the inference that the reduction of PhA reflects (and is caused by) an inflammatory state [16].

Studies correlating PhA values with levels of inflammatory markers directly in athletes or, at least, in the context of regular training are limited. On the contrary, most of the available evidence derives from observations made on subjects with specific pathological conditions or subjects who are healthy but, in any case, not engaged in a training load that would allow us to consider them athletes. However, it must be remembered that the biological mechanisms underlying the deleterious effects of inflammation and oxidative stress on the integrity of cell membranes, hence on that of tissues and, therefore, on the general state of health are the same. Therefore, it is possible the information derived from these studies can be transferred to the sport field, suitably contextualised, and used to understand (or, at least, hypothesise) the biological and physiological variations that may occur during exercise.

#### RT-related inflammation and its potential implication on PhA

Among the various kind of sport, RT is strongly linked to OT risk as it can cause acute fatigue due to reduced neuromuscular activation and sequencing, manifested as a short-term performance decrease (seconds to hours) due to impairment of central and/or peripheral mechanisms. A single overloading RT session, however, results in acute fatigue and a temporary performance decrease, while short-term accumulated training above the habitual level followed by recovery can lead to a supercompensation (F-OR), or a diminished adaptive response and long-term performance decrement (nF-OR). Prolonged exposure to such training may lead to OTS [13]. Implementing short-term OR is common in strength sports, where a high-volume/high-intensity RT over a 2–4-week period often leads to F-OR due to improved performance. Various performance, neuroendocrine, neuromuscular, and biochemical markers have been proposed to determine nF-OR/OTS in strength sports and RT, but no single test or method has pinpointed when F-OR transitions to nF-OR or OTS. A dose–response transition from F-OR to nF-OR might exist, identifiable through physiological markers or performance testing, but current literature has not identified this [20]. Evidence suggests that nF-OR is a real consequence of excessive and chronic RT without sufficient recovery, particularly in extreme conditioning practices [20].

RT has also an effect on the inflammatory state. As previously reported in the literature, in fact, certain periods of RT can lead to an increase in the release of proinflammatory cytokines such as interleukin-6 (IL-6) by the muscle following motor unit contractions, and this effect would appear to be related, at least in part, to the reduction in muscle glycogen levels [96]. This is due to the specific characteristics of this type of training. In general, RT consists of performing static or dynamic muscle contractions against a resistance of different intensity. Various types of muscle actions can be identified during training, such as (i) isometric actions (which are characterised by a variation in muscle tension, without a variation in length) [97], (ii) concentric (which occur when the muscle tension is such that it exceeds the magnitude of the external load, resulting in contraction and shortening of the muscle) [98] and (iii) eccentric (which occur when the contraction force is less than the external resistance, resulting in lengthening of the muscle). The latter action, during RT is responsible for muscle damage that occurs to a greater extent than concentric [96]. The physiological response to tissue damage is inflammation, which manifests itself in the synthesis and release of cytokines, and which varies depending on the type, intensity and duration of exercise, as well as recovery time and training status [96]. Although it may appear schematic and mechanistic, however, the intricate role played by cytokines in a specific context such as RT may not be easy to understand. It should be emphasised, in fact, that regardless of the pro-inflammatory nature of some cytokines (such as IL-1β, IL-8 and TNF-α) and the anti-inflammatory nature of others (such as IL-1ra and IL-10), there is sometimes a bi-directional relationship between one and the other, which is established with a homeostatic purpose as a consequence of training [96]. More specifically, it has been reported that the release of IL-6 (with its known pro-inflammatory action) by the muscle represents a stimulus for the secretion of anti-inflammatory cytokines, such as IL-1ra and IL-10, and, at the same time, it inhibits the release of IL-1β and TNF-α, thus suggesting an anti-inflammatory role of IL-6 secreted by myocytes in response to exercise [96].

Another important aspect to take into account concerns the physiological response to the different timing with which training is performed. It appears, in fact, that RT elicits two diametrically opposed responses in acute and chronic. In acute, RT leads to increased synthesis and release of ROS, which is reflected in a concomitant increase in circulating levels of pro-inflammatory cytokines. On the contrary, in chronic these phenomena are reversed, and an increase in cellular antioxidant capacity is observed, as well as a reduction in the levels of markers of inflammation, due to a mechanism of adaptation of the body to training. Interestingly, these long-term effects are observed both in response to exercise and in resting conditions and are more pronounced in trained subjects than in those who are not [96].

On the basis of what we have described so far, therefore, the effect of RT on oxidative state and inflammatory response appears clear, paving the way towards possible hypotheses and speculations regarding the use of instruments and/or parameters capable of monitoring these changes in real time, without the need for invasive, costly or, at the least, impractical application. Among these, the use of raw BIA parameters may appear inspiring, particularly PhA which, as extensively described in the previous section, has been validated as a surrogate marker of systemic inflammation and oxidative stress in several studies. Nevertheless, studies that have investigated the possible correlation between the inflammatory state induced by RT and related changes in PhA are limited, particularly on athletes or, at least, regularly trained subjects, probably due to the difficulty of designing ad hoc protocols. At the moment, therefore, only speculations on a theoretical basis are possible in sport, suggesting a potential increase in PhA values (in chronic) that would reflect the demonstrated anti-inflammatory effect of training. In support of this thesis, a study evaluated, in elderly women, the effects of a RT protocol on PhA while monitoring markers of inflammation and oxidative stress, observing contextual improvements in both PhA values and marker levels of inflammation [90].

In sports, it is well understood that regular and thorough assessments of an athlete’s overall health are essential for early detection of any alterations that could compromise performance. Therefore, understanding changes in raw BIA parameters is just as crucial in acute conditions as it is in chronic ones. Therefore, some questions remain unanswered at present: *considering the pro-inflammatory action described in acute*,* how does PhA vary following a single RT session? Does it actually decrease*,* which could correlate with a concomitant increase in circulating levels of inflammatory markers*,* as might be theorised?* This type of information would be particularly useful both to monitor the athlete’s health status and to optimally target the nutritional intervention and training scheme in order to prevent damage or negatively impact performance.

### Is PhA related to overtraining? Conclusions, hypothesis, and future perspectives

This narrative review deals with an in-depth examination of the well-established use of BIA in sport, emphasising how, beyond its mere use for the assessment of BC, this method can provide important information capable of highlighting and describing biological variations (physiological and otherwise) to external stimuli, such as exercise. We are referring, in this case, to the raw BIA parameters that describe the bioelectric properties of the body, and which are subject to variations that reflect the aforementioned biological responses. Of these, the focus of this article has been placed on PhA, which has been shown to best reflect the cellular response to training, as well as being validated as a surrogate marker of inflammatory and oxidative states. In the sport context, therefore, a careful and periodic assessment of PhA allows nutritionists and coaches to monitor the progress of their respective interventions on the athlete and, if necessary, optimise them in order to ensure performance. This concept takes on further relevance when considering the described effect of certain types of training (such as RT) on the inflammatory response, which varies in acute and chronic, reflecting a long-term improvement in PhA values. This (anti-inflammatory) effect of RT observed in chronic is, inevitably, to be related to the regularity of the training (in terms of intensity, duration and frequency), as described by Otsuka’s observations, who reported a significant increase in PhA after 12 weeks of moderate-intensity RT, which, on the other hand, was not observed in the group following low-intensity RT [69]. Exercise intensity, therefore, as a component of training volume, is an important determinant to take into account in the logic of ‘regularity’, which inevitably results in good athletic performance.

**Fig. 1:**
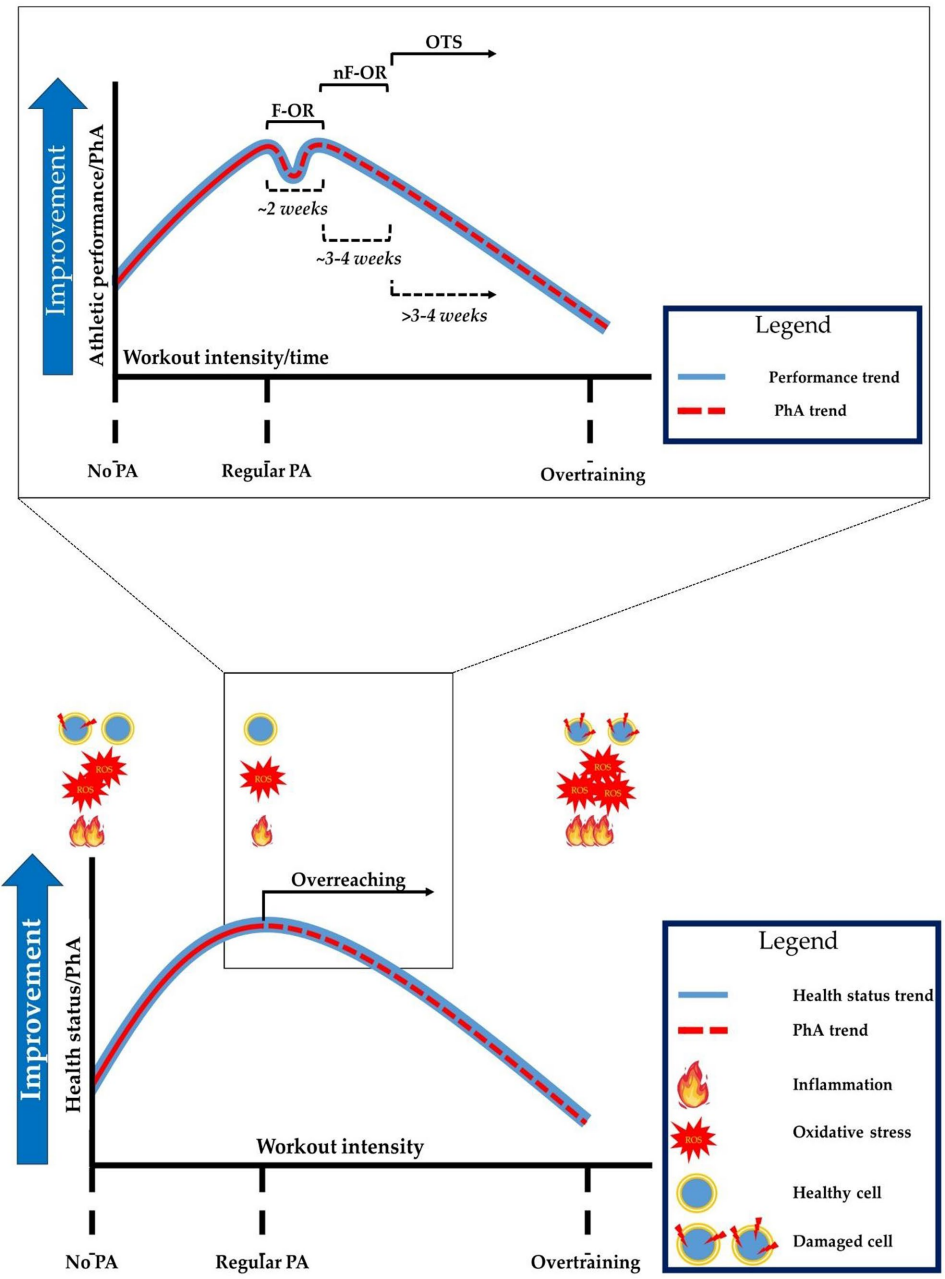
The potential use of PhA in overtraining identification

In the context of a holistic view, therefore, the two-way relationship between state of health and training would seem to follow a linear trend where, as the volume of training increases, an improvement in the state of well-being is observed. This concept may be true to a certain extent, as the body’s physiological response to training also follows a homeostatic model, in which equilibrium is achieved by the principle of regularity. This is the case with the OTS experienced by some athletes engaged in excessive training volumes. OTS manifests itself with important repercussions on the state of health, ranging from mood alterations to physiological and biochemical ones [13]. These alterations are the result of excessive musculoskeletal stress, associated with an insufficient rest and recovery period that induces an acute inflammatory response that can become chronic over time. Thus, systemic inflammation can occur due to the synthesis and secretion of large amounts of the pro-inflammatory cytokines IL-1β, IL-6, and TNF- α [99]. This concept, therefore, lays the foundations for a re-evaluation of the relationship between health status and training, which would appear not to be described by a linear trend, but rather subtended by a bell-shaped curve in which, following the hormesis model, health status improves as the training volume increases until a threshold value is reached, beyond which it tends to worsen (Fig. 1).

Considering that, as widely discussed, health status and inflammation are associated with PhA values, with regard to training, we ask the following question: *although a chronic anti-inflammatory effect has been described*,* which coincides with an increase in PhA levels*,* what happens when the intensity or*,* at least*,* the volume of training exceeds a certain threshold of individual tolerance of the organism*,* thus pushing towards OR or OT?* The assumptions in this paper would suggest a predictable reduction in PhA values, which would justify an increased inflammatory response. *But would these possible reductions be due to changes in its resistive (R) or capacitive (Xc) component?* To answer these questions, at least in part, it is possible to refer to the hormetic principle that would describe the risk of developing OTS. From an integrated perspective based on the rational use of BIA in sport, PhA can share with health status the positioning on the y-axis of this hypothetical curve (Fig. 1). This would be justified by the previously reported observations concerning changes in PhA as a function of both training intensity and inflammation marker values. From analysis and interpretation of this graphic representation, therefore, we propose (at least on a theoretical basis) the possible use of PhA as a prognostic marker of OTS risk. According to our theory, then, the observation of a reduction in PhA values, in the absence of abnormal changes in hydration status but in the presence of alterations in intra-/extracellular fluid distribution described by significant increases in R and/or reductions in Xc, could be indicative of impaired cellular integrity due to maximal stress and training overload, as previously reviewed [42]. Therefore, on this basis, a sudden reduction in PhA during training (particularly if preceded over time by increases in its value) could indicate the exceeding of the hormetic threshold, thus, the possible establishment of an OTS.

As described in the previous sections, the currently available literature dictates the possibility of purely speculative conclusions, given the absence of specific studies. In particular, the gaps in the literature in this regard relate to (i) specificity of the population (which must be represented by athletes), (ii) identification of OR (both F-OR and nF-OR), OT, and OTS both under experimental conditions or in the real-setting of the competitive season, and (iii) use of validated instrumentation and protocols. The existence of these gaps, therefore, implies the need for future longitudinal studies. Specifically, there is an undeniable need for targeted studies to confirm or challenge our theory, which would provide valuable insights not only for early detection of OTS risk but also for guiding appropriate adjustments in nutrition (including diet and potentially supplementation) and training. Such research would enable the adoption of personalized strategies to manage existing or emerging biological alterations. These studies could be designed to determine the optimal magnitude of PhA’s change necessary to induce adaptations in body composition and performance without allowing acute fatigue to progress into chronic fatigue. Moreover, changes in PhA during acute fatigue should be studied in relation to their potential dependence on both central and peripheral factors. Therefore, the primary aim could be to evaluate the association between changes in PhA and inflammatory markers typically linked to fatigue. Additionally, longitudinal studies could incorporate performance measures (such as sprints or jumps) to assess how their decline during phases of acute fatigue is associated with changes in PhA.

If PhA’s role as a potential biomarker for monitoring fatigue and preventing OR from developing into OT is confirmed, the possible applications could be numerous. For example, in sports that involve a preparatory phase, it could be used to manage training load by identifying the optimal percentage of PhA decrease that triggers subsequent adaptations aimed at improving performance. In this case, internal load could be monitored through PhA, allowing external load to be adjusted accordingly, both at a group and individual level. Interestingly, a state of inflammation, typical of fatigue or certain types of injuries, can lead to extracellular fluid accumulation (hyperhydration), which can be detected through reductions in Z as well as in PhA. Consequently, by combining PhA with vector length assessment within the R-Xc graph, specific zones of vector displacement could be identified for each sport during these phases. These zones would help pinpoint risky areas where the vector’s position could indicate chronic fatigue, characteristic of OT. On the contrary, defining optimal patterns of change could guide the staff toward selecting ideal training loads. This approach, not explored in depth in this narrative review, is known as BIVA and could be incorporated into the proposed investigations. Alternatively, during competition, PhA monitoring could help plan recovery phases after training sessions or events, depending on the extent of its decrease. Lastly, in RT, assessing changes in PhA could provide valuable insights into the supercompensation process, thereby helping to manage the scheduling of future training sessions, as well as supplementation and nutrition during recovery.

As a corollary to what has been described and hypothesized so far, it should be emphasized that although PhA can be a valuable tool for early identification of OTS, its integration with other parameters and/or techniques can allow for more in-depth monitoring of the athlete. This concept is not new. In fact, already in other clinical settings, the use of PhA in combination with other techniques (including tissue ultrasound) or parameters (such as hand grip strength, HGS) is suggested for a comprehensive and functional assessment of the subject [100]. This multifactorial and multidisciplinary view is supported by evidence of correlations between PhA and such parameters [101], particularly HGS [17], also reported in athletes [102], which allows consolidation of the concept of PhA as a marker of muscle quality. At the same time, however, this evidence suggests the importance of integrating these parameters into the overall assessment of the athlete, considering that the observation of negative changes in PhA contextual to those in HSG may allow for more accurate indications of a possible onset of OTS. Again, however, longitudinal studies on the athletic population, appropriately designed, are necessary in order to obtain both information about physiological (or non-physiological) changes in these parameters at various stages of athletic training, but also to identify new indices given by the combination of BIA parameters and other techniques, such as ultrasonography and dynamometry.

## Electronic supplementary material

Below is the link to the electronic supplementary material.

Supplementary Material 1

## Data Availability

Not applicable.

## Abbreviations

BIA: Bioelectrical impedance analysis
BIVA: Bioelectrical impedance vector analysis
Z: Impedance
R: Resistance
Xc: Reactance
PhA: Phase angle
TBW: Total body water
ICW: Intracellular body water
ECW: Extracellular body water
BC: Body composition
BCM: Body cell mass
OTS: Overtraining Syndrome
OT: Overtraining
OR: Overreaching
RT: Resistance training
PAL: Physical activity levels
FFM: Fat-free mass
L-BIA: Localised phase-sensitive BIA
HRF: Health-related fitness
F-OR: Functional overreaching
nF-OR: Non-functional overreaching
ROS: Reactive oxygen species

## References

[1] Lukaski H, Raymond-Pope CJ. New frontiers of body composition in Sport. Int J Sports Med. 2021;42(7):588–601. 33621995 10.1055/a-1373-5881 PMC8421000

[2] Ward LC, Brantlov S. Bioimpedance basics and phase angle fundamentals. Rev Endocr Metab Disord. 2023;24(3):381–91. 36749540 10.1007/s11154-022-09780-3 PMC10140124

[3] Moon JR. Body composition in athletes and sports nutrition: an examination of the bioimpedance analysis technique. Eur J Clin Nutr. 2013;67(Suppl 1):S54–9. 23299872 10.1038/ejcn.2012.165

[4] Campa F, Coratella G, Cerullo G, Noriega Z, Francisco R, Charrier D, et al. High-standard predictive equations for estimating body composition using bioelectrical impedance analysis: a systematic review. J Transl Med. 2024;22:515. 38812005 10.1186/s12967-024-05272-x PMC11137940

[5] Piccoli A, Piazza P, Noventa D, Pillon L, Zaccaria M. A new method for monitoring body fluid variation by bioimpedance analysis: the RXc graph. Med sci Sport Exerc. 1996;28(12):1517–22. 10.1097/00005768-199612000-00012 8970147

[6] Ng JK, Lau SL, Chan GC, Tian N, Li PK. Nutritional assessments by Bioimpedance Technique in Dialysis patients. Nutrients. 2023;16(1):15. 38201845 10.3390/nu16010015 PMC10780416

[7] Kyle UG, Bosaeus I, De Lorenzo AD, Deurenberg P, Elia M, Gómez JM, Heitmann BL, Kent-Smith L, Melchior JC, Pirlich M, Scharfetter H, Schols AM, Pichard C. C of the EWG. Bioelectrical impedance analysis–part I: review of principles and methods. Clin Nutr. 2004;23(5):1226–43. 15380917 10.1016/j.clnu.2004.06.004

[8] Mulasi U, Kuchnia AJ, Cole AJ, Earthman CP. Bioimpedance at the bedside: current applications, limitations, and opportunities. Nutr Clin Pract. 2015;30(2):180–93. 25613832 10.1177/0884533614568155

[9] Sergi G, De Rui M, Stubbs B, Veronese N, Manzato E. Measurement of lean body mass using bioelectrical impedance analysis: a consideration of the pros and cons. Aging Clin Exp Res 29AD;4(591–7). 10.1007/s40520-016-0622-6 27568020

[10] Kyle UG, Bosaeus I, De Lorenzo AD, Deurenberg P, Elia M, Manuel Gómez J, Heitmann L, Kent-Smith B, Melchior L, Pirlich JC, Scharfetter M, Schols HMWJ, Pichard A. Bioelectrical impedance analysis-part II: utilization in clinical practice. Clin Nutr. 2004;23(6):1430–53. 15556267 10.1016/j.clnu.2004.09.012

[11] Di Vincenzo O, Marra M, Scalfi L. Bioelectrical impedance phase angle in sport: a systematic review. J Int Soc Sports Nutr. 2019;16(1):49. 31694665 10.1186/s12970-019-0319-2 PMC6833254

[12] Campa F, Thomas DM, Watts K, Clark N, Baller D, Morin T, et al. Reference percentiles for Bioelectrical Phase Angle in athletes. Biology (Basel). 2022;2022:264. 10.3390/biology11020264 PMC8869633 35205130

[13] Meeusen R, Duclos M, Foster C, Fry A, Gleeson M, Nieman D, Raglin J, Rietjens G, Steinacker J, Urhausen A. European College of Sport Science & AC of SM. Prevention, diagnosis, and treatment of the overtraining syndrome: joint consensus statement of the European College of Sport Science and the American College of Sports Medicine. Med Sci Sports Exerc. 2013;45(1):186–205. 23247672 10.1249/MSS.0b013e318279a10a

[14] Sardinha LB, Rosa GB. Phase angle, muscle tissue, and resistance training. Rev Endocr Metab Disord. 2023;24(3):393–414. 36759377 10.1007/s11154-023-09791-8 PMC10140117

[15] Custódio Martins P, de Lima TR, Silva AM, Santos Silva DA. Association of phase angle with muscle strength and aerobic fitness in different populations: a systematic review. Nutrition. 2022;93:111489. 34688022 10.1016/j.nut.2021.111489

[16] da Silva BR, Orsso CE, Gonzalez MC, Sicchieri JMF, Mialich MS, Jordao AA, Prado CM, da Silva BR, Orsso CE, Gonzalez MC, Sicchieri JMF, Mialich MS, et al. Phase angle and cellular health: inflammation and oxidative damage. Rev Endocr Metab Disord. 2023;24(3):543–62. 36474107 10.1007/s11154-022-09775-0 PMC9735064

[17] Cirillo E, Pompeo A, Cirillo FT, Vilaça-Alves J, Costa P, Ramirez-Campillo R, Dourado AC, Afonso J, Casanova F. Relationship between Bioelectrical Impedance Phase Angle and Upper and Lower Limb muscle strength in athletes from several sports: a systematic review with Meta-analysis. Sports. 2023;11:107. 37234063 10.3390/sports11050107 PMC10222561

[18] Nescolarde L, Talluri A, Yanguas · Javier, Lukaski H. Phase angle in localized bioimpedance measurements to assess and monitor muscle injury. Rev Endocr Metab Disord. 2023;24:415–28. 36847994 10.1007/s11154-023-09790-9 PMC10140135

[19] Rosa GB, Hetherington-Rauth M, Magalhães JP, Correia IR, Bernardino AVSL. Limb-specific isometric and isokinetic strength in adults: the potential role of regional bioelectrical impedance analysis-derived phase angle. Clin Nutr. 2024;43(1):154–62. 38048645 10.1016/j.clnu.2023.11.039

[20] Bell L, Ruddock A, Maden-Wilkinson T, Rogerson D. Overreaching and overtraining in strength sports and resistance training: a scoping review. J Sports Sci. 2020;38(16):1897–912. 32602418 10.1080/02640414.2020.1763077

[21] Fry AC, Kraemer WJ. Resistance Exercise Overtraining and Overreaching. Sports Med. 1997;23(2):106–29. 9068095 10.2165/00007256-199723020-00004

[22] Mundstock E, Amaral MA, Baptista RR, Sarria EE, Santos D, Filho RRG, Rodrigues AD, Forte CAS, Castro GC, Padoin L, Stein AV, Perez R, Ziegelmann LM, P. K., Mattiello R. Association between phase angle from bioelectrical impedance analysis and level of physical activity: systematic review and meta-analysis. Clin Nutr. 2019;38(4):1504–10. 30224304 10.1016/j.clnu.2018.08.031

[23] Ribeiro AS, Schoenfeld BJ, Souza MF, Tomeleri CM, Silva AM, Teixeira DC, Sardinha LB, Cyrino ES. Resistance training prescription with different load-management methods improves phase angle in older women. Eur J Sport Sci. 2017;17(7):913–21. 28394730 10.1080/17461391.2017.1310932

[24] Gupta MS. Georg Simon Ohm and Ohm’s Law. In: IEEE Trans Educ. 1980. pp. 156–62.

[25] Paoli A, Campa F. Problems and opportunities in the use of Bioelectrical Impedance Analysis for assessing body composition during ketogenic diets: a scoping review. Curr Obes Rep. 1234;13:496–509. 10.1007/s13679-024-00573-0 PMC11306364 38802722

[26] Baumgartner RN, Chumlea WC, Roche AF. Estimation of body composition from bioelectric impedance of body segments. he Am J Clin Nutr. 1989;50(2):221–6. 10.1093/ajcn/50.2.221 2756908

[27] Norman K, Stobäus N, Pirlich M, Bosy-Westphal A. Bioelectrical phase angle and impedance vector analysis–clinical relevance and applicability of impedance parameters. Clin Nutr. 2012;31(6):854–61. 22698802 10.1016/j.clnu.2012.05.008

[28] Gonzalez MC, Barbosa-Silva TG, Bielemann RM, Gallagher D, Heymsfield SB. Phase angle and its determinants in healthy subjects: influence of body composition. Am J Clin Nutr. 2016;103(3):712–6. 26843156 10.3945/ajcn.115.116772 PMC6546229

[29] Marini E, Campa F, Buffa R, Stagi S, Matias CN, Toselli S, et al. Phase angle and bioelectrical impedance vector analysis in the evaluation of body composition in athletes. Clin Nutr. 2020;39(2):447–54. 30850270 10.1016/j.clnu.2019.02.016

[30] Baumgartner RN, Chumlea WC, Roche AF. Bioelectric impedance phase angle and body composition. Am J Clin Nutr. 1998;48(1):16–23. 10.1093/ajcn/48.1.16 3389323

[31] O’Brien C, Young AJ, Sawka MN. Bioelectrical impedance to estimate changes in hydration status. Int J Sports Med. 2002;23(5):361–6. 12165888 10.1055/s-2002-33145

[32] Piccoli A, Rossi B, Pillon L, Bucciante G. A new method for monitoring body fluid variation by bioimpedance analysis: the RXc graph. Kidney Int. 1994;46(2):534–9. 7967368 10.1038/ki.1994.305

[33] Buffa R, Saragat B, Cabras S, Rinaldi AC, Marini E. Accuracy of specific BIVA for the assessment of body composition in the United States population. PLoS ONE. 2013;8(3):e58533. 23484033 10.1371/journal.pone.0058533 PMC3590169

[34] Marini E, Sergi G, Succa V, Saragat B, Sarti S, Coin A, Manzato E, Buffa R. Efficacy of specific bioelectrical impedance vector analysis (BIVA) for assessing body composition in the elderly. J Nutr Health Aging. 2013;17(6):515–21.23732547 10.1007/s12603-012-0411-7

[35] Gatterer H, Schenk K, Laninschegg L, Schlemmer P, Lukaski H, Burtscher M. Bioimpedance identifies body fluid loss after Exercise in the heat: A Pilot Study with Body Cooling. 10.1371/journal.pone.0109729 PMC4184898 25279660

[36] Mala L, Maly T, Zahalka F, Dragijsky M. Changes in body composition due to weight reduction by elite youth judo athletes in short period pre-competition. Arch Budo Sci Martial Arts Extrem Sport. 2016;12:197–203.

[37] Lukaski HCTA. Phase angle as an index of physiological status: validating bioelectrical assessments of hydration and cell mass in health and disease. Rev Endocr Metab Disord. 2023;24(3):371–9. 36336754 10.1007/s11154-022-09764-3

[38] Yamaguchi S, Inami T, Ishida H, Nagata N, Murayama M, Morito A, Yamada SKN. Bioimpedance analysis for identifying new indicators of exercise–induced muscle damage. Sci Rep. 2024;14(1):15299. 38961243 10.1038/s41598-024-66089-8 PMC11222495

[39] Cupisti A, Capitanini A, Betti G, D’Alessandro C, Barsotti G. Assessment of habitual physical activity and energy expenditure in dialysis patients and relationships to nutritional parameters. Clin Nephrol. 2011;75(3):218–25. 21329632 10.5414/cnp75218

[40] Jungblut SA, Frickmann H, Zimmermann B, Müller U, Bargon J. The effects of physical training on the body composition of patients with COPD. Pneumologie. 2009;63(7):374–9. 19475523 10.1055/s-0029-1214713

[41] Barbosa-Silva MC, Barros AJ. Bioelectrical impedance analysis in clinical practice: a new perspective on its use beyond body composition equations. Curr Opin Clin Nutr Metab Care. 2005;8(3):311–7. 15809535 10.1097/01.mco.0000165011.69943.39

[42] Martins PC, Moraes MS, Silva DAS. Cell integrity indicators assessed by bioelectrical impedance: a systematic review of studies involving athletes. J Bodyw Mov Ther. 2020;24(1):154–64. 31987537 10.1016/j.jbmt.2019.05.017

[43] Kumar S, Dutt A, Hemraj S, Bhat S, Manipadybhima B. Phase angle measurement in healthy human subjects through bio-impedance analysis. Iran J Basic Med Sci. 2012;15(6):1180–4. 23653848 PMC3646229

[44] Buffa R, Floris G, Marini E. Migration of the bioelectrical impedance vector in healthy elderly subjects. Nutrition. 2003;19(11–12):917–21. 14624938 10.1016/s0899-9007(03)00180-1

[45] Ribeiro AS, Nascimento MA, Schoenfeld BJ, Nunes JP, Aguiar AF, Cavalcante EF, Silva AM, Sardinha LB, Fleck SJ, Cyrino ES. Effects of single set resistance training with different frequencies on a cellular health indicator in older women. J Aging Phys Act. 2018;26(4):537–43. 29182426 10.1123/japa.2017-0258

[46] Lukaski HC. Evolution of bioimpedance: a circuitous journey from estimation of physiological function to assessment of body composition and a return to clinical research. Eur J Clin Nutr. 2013;67(Suppl 1):S2–9. 23299867 10.1038/ejcn.2012.149

[47] Marques A, Henriques-Neto D, Peralta M, Martins J, Gomes F, Popovic S, Masanovic B, Demetriou Y, Schlund A, Ihle A. Field-Based Health-related physical fitness tests in children and adolescents: a systematic review. Front Pediatr. 2021;9:640028. 33748047 10.3389/fped.2021.640028 PMC7973114

[48] Ballarin G, Valerio G, Alicante P, Di Vincenzo O, Monfrecola F, Scalfi L. Could BIA-derived phase angle predict health-related musculoskeletal fitness? A cross-sectional study in young adults. Nutrition. 2024;122:112388. 38442652 10.1016/j.nut.2024.112388

[49] Mann S, Wade M, Fisher J, Giessing J, Gentil P, Steele J. Phase Angle as an Indicator of Health and Fitness in patients entering an Exercise Referral Scheme. J Am Med Dir Assoc. 2018;19(9):809–10. 30029932 10.1016/j.jamda.2018.06.005

[50] Perez AC, Cabral de Oliveira AC, Estevez E, Molina AJ, Prieto JG, Alvarez AI. Mitochondrial, sarcoplasmic membrane integrity and protein degradation in heart and skeletal muscle in exercised rats. Comp Biochem Physiol C Toxicol Pharmacol. 2003;134(2):199–206. 12600679 10.1016/s1532-0456(02)00247-8

[51] Cunanan AJ, DeWeese BH, Wagle JP, Carroll KM, Sausaman R, Hornsby WG 3rd, Haff GG, Triplett NT, Pierce KC, Stone MH. The general adaptation syndrome: a foundation for the concept of periodization. Ports Med (Auckland NZ). 2018;48(4):787–97. 10.1007/s40279-017-0855-3 29307100

[52] Issurin VB. Generalized training effects induced by athletic preparation. A review. J Sport Med Phys Fit. 2009;49(4):333–45. 20087292

[53] Nabuco HCG, Silva AM, Sardinha LB, Rodrigues FB, Tomeleri CM, Ravagnani FCP, et al. Phase Angle is moderately Associated with short-term maximal intensity efforts in Soccer players. Int J Sports Med. 2019;40(11):739–43. 31437860 10.1055/a-0969-2003

[54] Reis JF, Matias CN, Campa F, Morgado JP, Franco P, Quaresma P, et al. Bioimpedance Vector patterns changes in response to Swimming Training: An Ecological Approach. Int J Environ Res Public Heal. 2020;17:4851. 10.3390/ijerph17134851 PMC7369706 32640533

[55] Mascherini G, Gatterer H, Lukaski H, Burtscher MGG. Changes in hydration, body-cell mass and endurance performance of professional soccer players through a competitive season. J Sport Med Phys Fit. 2015;55(7–8):749–7555. 25303072

[56] American College of Sports Medicine. American College of Sports Medicine position stand. Progression models in resistance training for healthy adults. Med Sci Sports Exerc. 2009;41(3):687–708. 19204579 10.1249/MSS.0b013e3181915670

[57] Taniguchi M, Yamada Y, Ichihashi N. Acute effect of multiple sets of fatiguing resistance exercise on muscle thickness, echo intensity, and extracellular-to-intracellular water ratio. Appl Physiol Nutr Metab. 2020;45(2):213–9. 31299164 10.1139/apnm-2018-0813

[58] Hirono T, Ikezoe T, Taniguchi M, Tanaka H, Saeki J, Yagi M, Umehara J, Ichihashi N. Relationship between muscle swelling and Hypertrophy Induced by Resistance Training. J Strength Cond Res. 2022;36(2):359–64. 31904714 10.1519/JSC.0000000000003478

[59] Chan ST, Johnson AW, Moore MH, Kapadia CR, Dudley HA. Early weight gain and glycogen-obligated water during nutritional rehabilitation. Hum Nutr Clin Nutr. 1982;36(3):223–32. 6811511

[60] Piccoli A, Pastori G, Codognotto M, Paoli A. Equivalence of information from single frequency v. bioimpedance spectroscopy in bodybuilders. Br J Nutr. 2007;97(1):182–92. 17217575 10.1017/S0007114507243077

[61] Souza MF, Tomeleri CM, Ribeiro AS, Schoenfeld BJ, Silva AM, Sardinha LB, Cyrino ES. Effect of resistance training on phase angle in older women: a randomized controlled trial. Scand J Med Sci Sports. 2017;27(11):1308–16. 27541287 10.1111/sms.12745

[62] Ribeiro AS, Avelar A, Dos Santos L, Silva AM, Gobbo LA, Schoenfeld BJ, Sardinha LB, Cyrino ES. Hypertrophy-type resistance training improves Phase Angle in Young Adult men and women. Int J Sports Med. 2017;38(1):35–40. 27793064 10.1055/s-0042-102788

[63] MacDougall JD, Ward GR, Sale DG, Sutton JR. Biochemical adaptation of human skeletal muscle to heavy resistance training and immobilization. J Appl Physiol. 1977;43(4):700–3. 908686 10.1152/jappl.1977.43.4.700

[64] Frigeri A, Nicchia GP, Verbavatz JM, Valenti G, Svelto M. Expression of aquaporin-4 in fast-twitch fibers of mammalian skeletal muscle. J Clin Invest. 1998;102(4):695–703. 9710437 10.1172/JCI2545 PMC508931

[65] Schoenfeld BJ. Does exercise-induced muscle damage play a role in skeletal muscle hypertrophy? J Strength Cond Res. 2012;26(5):1441–53. 22344059 10.1519/JSC.0b013e31824f207e

[66] Schoenfeld BJ. Potential mechanisms for a role of metabolic stress in hypertrophic adaptations to resistance training. Sports Med. 2013;43(3):179–94. 23338987 10.1007/s40279-013-0017-1

[67] Dangott B, Schultz E, Mozdziak PE. Dietary creatine monohydrate supplementation increases satellite cell mitotic activity during compensatory hypertrophy. Int J Sports Med. 2000;21(1):13–13. 10683092 10.1055/s-2000-8848

[68] Huemer MT, Petrera A, Hauck SM, Drey M, Peters ATB. Proteomics of the phase angle: results from the population-based KORA S4 study. Clin Nutr. 2022;41(8):1818–26. 35834914 10.1016/j.clnu.2022.06.038

[69] Otsuka Y, Yamada Y, Maeda A, Izumo T, Rogi T, Shibata H, Fukuda M, Arimitsu T, Miyamoto N, Hashimoto T. Effects of resistance training intensity on muscle quantity/quality in middle-aged and older people: a randomized controlled trial. J Cachexia Sarcopenia Muscle. 2022;13(2):894–908. 35187867 10.1002/jcsm.12941 PMC8977953

[70] Carrard J, Rigort AC, Appenzeller-Herzog C, Colledge F, Königstein K, Hinrichs T. Schmidt-Trucksäss A. Diagnosing Overtraining Syndrome: a scoping review. Sports Health. 2022;14(5):665–73. 34496702 10.1177/19417381211044739 PMC9460078

[71] Cadegiani FA, da Silva PHL, Abrao TCPKC. Diagnosis of Overtraining Syndrome: results of the endocrine and metabolic responses on Overtraining Syndrome Study: EROS-DIAGNOSIS. J Sport Med (Hindawi Publ Corp). 2020;2020:3937819. 10.1155/2020/3937819 PMC7193300 32373644

[72] Cadegiani FAKC. Novel causes and consequences of overtraining syndrome: the EROS-DISRUPTORS study. BMC Sport Sci Med Rehabil. 2019;11:21. 10.1186/s13102-019-0132-x PMC6751688 31548891

[73] Mallardo M, Daniele A, Musumeci GNE. A narrative review on adipose tissue and overtraining: shedding light on the interplay among adipokines, Exercise and Overtraining. Int J Mol Sci. 2024;25(7):4089. 38612899 10.3390/ijms25074089 PMC11012884

[74] Finaud J, Lac G, Filaire E. Oxidative stress: relationship with exercise and training. Sports Med. 2006;36(4):327–58. 16573358 10.2165/00007256-200636040-00004

[75] Tiidus PM. Radical species in inflammation and overtraining. Can J Physiol Pharmacol. 1998;76(5):533–8. 9839079 10.1139/cjpp-76-5-533

[76] König D, Wagner KH, Elmadfa I, Berg A. Exercise and oxidative stress: significance of antioxidants with reference to inflammatory, muscular, and systemic stress. Exerc Immunol Rev. 2001;7:108–33. 11579745

[77] Steinacker JM, Lormes W, Reissnecker S, Liu Y. New aspects of the hormone and cytokine response to training. Eur J Appl Physiol. 2004;91(4):382–91. 14608461 10.1007/s00421-003-0960-x

[78] Luti S, Modesti A, Modesti PA, Inflammation. Peripheral Signals and Redox Homeostasis in Athletes Who Practice Different Sports. ntioxidants (Basel, Switzerland). 2020;9(11):1065. 10.3390/antiox9111065 PMC7693221 33143147

[79] Radak Z, Chung HY, Koltai E, Taylor AW. Exercise, oxidative stress and hormesis. Ageing Res Rev. 2008;7(1):34–42. 17869589 10.1016/j.arr.2007.04.004

[80] Sproston NR, Ashworth JJ. Role of C-reactive protein at sites of inflammation and infection. Front Immunol. 2018;9:754. 29706967 10.3389/fimmu.2018.00754 PMC5908901

[81] Planz B, Wolff JM, Gutersohn A, Stampfer DS, Jakse G. C-reactive protein as a marker for tissue damage in patients undergoing ESWL with or without retrograde stone manipulation. Urol Int. 1997;59(3):174–6. 9428435 10.1159/000283056

[82] Lukaski HC, Kyle UG, Kondrup J. Assessment of adult malnutrition and prognosis with bioelectrical impedance analysis: phase angle and impedance ratio. Curr Opin Clin Nutr Metab Care. 2017;20(5):330–9. 28548972 10.1097/MCO.0000000000000387

[83] Barrea L, Muscogiuri G, Pugliese G, Laudisio D, de Alteriis G, Graziadio C, et al. Phase Angle as an Easy Diagnostic Tool of Meta-inflammation for the nutritionist. Nutrients. 2021;13(5):1446. 33923291 10.3390/nu13051446 PMC8145306

[84] Barrea L, Arnone A, Annunziata G, Muscogiuri G, Laudisio D, Salzano C, Pugliese G, Colao A, Savastano S. Adherence to the mediterranean diet, dietary patterns and body composition in women with polycystic ovary syndrome (PCOS). Nutrients. 2019;11(10):2278. 31547562 10.3390/nu11102278 PMC6836220

[85] Barrea L, Muscogiuri G, Laudisio D, Di Somma C, Salzano C, Pugliese G, de Alteriis G, Colao A, Savastano S. Phase angle: a possible biomarker to quantify inflammation in subjects with obesity and 25(OH)D deficiency. Nutrients. 2019;11(8):1747. 31362440 10.3390/nu11081747 PMC6723101

[86] Barrea L, Fabbrocini G, Annunziata G, Muscogiuri G, Donnarumma M, Marasca C, et al. Role of nutrition and adherence to the mediterranean diet in the multidisciplinary approach of hidradenitis suppurativa: evaluation of nutritional status and its association with severity of disease. Nutrients. 2019;11(1):57. 10.3390/nu11010057 PMC6356593 30597889

[87] Barrea L, Macchia PE, Di Somma C, Napolitano M, Balato A, Falco A, Savanelli MC, Balato N, Colao A, Savastano S. Bioelectrical phase angle and psoriasis: a novel association with psoriasis severity, quality of life and metabolic syndrome. J Transl Med. 2016;14(1):130. 27165166 10.1186/s12967-016-0889-6 PMC4863378

[88] Barrea L, Altieri B, Muscogiuri G, Laudisio D, Annunziata G, Colao A, Faggiano A, Savastano S. Impact of nutritional status on gastroenteropancreatic neuroendocrine tumors (GEP-NET) aggressiveness. Nutrients. 2018;10(12):1854. 30513732 10.3390/nu10121854 PMC6316835

[89] Hui D, Moore J, Park M, Liu D, Bruera E. Phase Angle and the diagnosis of Impending Death in patients with Advanced Cancer: preliminary findings. Oncologist. 2019;24(6):e365–73. 30352942 10.1634/theoncologist.2018-0288 PMC6656508

[90] Tomeleri CM, Ribeiro AS, Cavaglieri CR, Deminice R, Schoenfeld BJ, Schiavoni D, Dos Santos L, de Souza MF, Antunes M, Venturini D, Barbosa DS, Sardinha LB, Cyrino ES. Correlations between resistance training-induced changes on phase angle and biochemical markers in older women. Scand J Med Sci Sports. 2018;28(10):2173–82. 29858504 10.1111/sms.13232

[91] Barrea L, Muscogiuri G, Aprano S, Vetrani C, de Alteriis G, Varcamonti L, Verde L, et al. Phase angle as an easy diagnostic tool for the nutritionist in the evaluation of inflammatory changes during the active stage of a very low-calorie ketogenic diet. Int J Obes (Lond). 2022;46(9):1591–7. 35614205 10.1038/s41366-022-01152-w PMC9130054

[92] Barrea L, Caprio M, Camajani E, Verde L, Perrini S, Cignarelli A et al. Ketogenic nutritional therapy (KeNuT)-a multi-step dietary model with meal replacements for the management of obesity and its related metabolic disorders: a consensus statement from the working group of the Club of the Italian society of Endocrinology (SI. J Endocrinol Invest. 2024;Advance on. 10.1007/s40618-023-02258-2 PMC10904420 38238506

[93] Muscogiuri G, El Ghoch M, Colao A, Hassapidou M, Yumuk V, Busetto L, et al. European Guidelines for Obesity Management in adults with a very low-calorie ketogenic Diet: a systematic review and Meta-analysis. Obes Facts. 2021;14(2):222–45. 33882506 10.1159/000515381 PMC8138199

[94] Muscogiuri G, Barrea L, Laudisio D, Pugliese G, Salzano C, Savastano S, et al. The management of very low-calorie ketogenic diet in obesity outpatient clinic: a practical guide. J Transl Med. 2019;17(1):356. 31665015 10.1186/s12967-019-2104-z PMC6820992

[95] Stobäus N, Pirlich M, Valentini L, Schulzke JD, Norman K. Determinants of bioelectrical phase angle in disease. Br J Nutr. 2012;107(8):1217–20. 22309898 10.1017/S0007114511004028

[96] Calle MC, Fernandez ML. Effects of resistance training on the inflammatory response. Nutr Res Pract. 2010;4(4):259–69. 20827340 10.4162/nrp.2010.4.4.259 PMC2933442

[97] Rosenblueth A., Alanis, J. & Rubio R. A comparative study of the isometric and isotonic contractions of striated muscles. Arch Int Physiol Biochim. 1958;66(3):330–53. 13560058 10.3109/13813455809084213

[98] Roig M, O’Brien K, Kirk G, Murray R, McKinnon P, Shadgan B, Reid WD. The effects of eccentric versus concentric resistance training on muscle strength and mass in healthy adults: a systematic review with meta-analysis. Br J Sports Med. 2009;43(8):556–68. 18981046 10.1136/bjsm.2008.051417

[99] Smith LL. Cytokine hypothesis of overtraining: a physiological adaptation to excessive stress? Med Sci Sports Exerc. 2000;32(2):317–31. 10694113 10.1097/00005768-200002000-00011

[100] García Almeida JM, García García C, Vegas Aguilar IM, Bellido Castañeda V. Bellido Guerrero D. Morphofunctional assessment of patient´s nutritional status: a global approach. Nutr Hosp. 2021;38(3):592–600. 33749304 10.20960/nh.03378

[101] Bellido D, García-García C, Talluri A, Lukaski HC, García-Almeida JM. Future lines of research on phase angle: strengths and limitations. Rev Endocr Metab Disord. 2023;24(3):563–83. 37043140 10.1007/s11154-023-09803-7 PMC10090740

[102] Hetherington-Rauth M, Leu CG, Júdice PB, Correia IR, Magalhães JPSL. Whole body and regional phase angle as indicators of muscular performance in athletes. Eur J Sport Sci. 2021;21(12):1684–92. 33280537 10.1080/17461391.2020.1858971

