# Supplementary material for: Use of phase angle as an indicator of overtraining in sport and physical training
Source: J Transl Med. 2024 Nov 29;22:1084. doi: 10.1186/s12967-024-05918-w (PMC11605935; doi:10.1186/s12967-024-05918-w)
Supplement: Supplementary file 1 — Supplementary Material 1 [file 12967_2024_5918_MOESM1_ESM.docx]

**Reviewer 1**

In this manuscript, the authors explore the application of Bioelectrical Impedance Analysis (BIA) in sports, highlighting an interesting focus on using this analysis for purposes beyond assessing body composition. Specifically, the authors examine the biological significance of the phase angle, hypothesizing that its variations over time may, in certain contexts, indicate the onset of overtraining. This insight represents an important advancement in understanding the complex world of sports and the body's responses to exercise stimuli. If confirmed, the author's hypothesis could provide a valuable tool for the early diagnosis of overtraining syndrome.

Overall, the article is well-structured. The complex mechanisms linking the physical principles of BIA to the body's physiological and pathological responses are well-described, and the biological justification for the proposed hypothesis is clearly presented.

Author’s response: We are very grateful to the Reviewer for his/her appreciation of our findings and his/her most positive report. We are hopeful that the constructive suggestions and the proposed corrections improved our manuscript. All the corrections in the revised manuscript are highlighted in yellow for the Reviewer's convenience.

Comments and Suggestions for Authors

As a suggestion, to enhance understanding for readers who may not be experts in the field, a more detailed description of the BIA parameters (possibly included outside the main text) could be beneficial.

Author’s response: We thank Reviewer#1 for this important suggestion. A table containing the biological significance of BIA raw parameters and their variations has been included in the revised version (table 1).

Out of personal curiosity (which could be addressed in the text), do the authors believe that only the phase angle can serve as an early marker for the onset of overtraining?

Author’s response: We thank Reviewer#1 for giving us the opportunity to address this interesting topic. Indeed, from a multifactorial and multidisciplinary perspective, it is not possible to think a single parameter as the exclusive marker of a complex condition such as OTS. On the contrary, the need to implement comprehensive evaluations, integrating multiple techniques and parameters, is becoming increasingly established in the literature. In this context, in our opinion, changes in PhA should be considered in combination with other parameters, including handgrip strength, or techniques, such as ultrasonography, in order to have a morphofunctional assessment, as previously described by other authors, with reference to other clinical contexts. Clearly, as this is not the focus of this article, the topic has not been extensively explored, but we found it important to mention it, as suggested.

As a minor point, a global check of the text to correct any typographical errors (e.g., use of abbreviations) is recommended.

Author’s response: We thank Reviewer#1. All typos (including the use of abbreviations) have been corrected.

In this referee's opinion, with these minor revisions, the article is suitable for publication.

**Reviewer 2**

The manuscript entitled "Use of Phase Angle as an Indicator of Overtraining in Sport and Physical Training" addresses an interesting and timely topic related to the use of Phase Angle (PhA) derived from Bioelectrical Impedance Analysis (BIA) as a potential biomarker for overtraining in sports. The review is comprehensive and explores the application of PhA beyond its traditional use in assessing body composition, suggesting its utility as an indicator of cellular health and overtraining status.

The manuscript is well-structured and covers relevant aspects of the topic; however, there are some critical areas that need to be addressed to enhance the scientific rigor and clarity of the review.

Author’s response: We are very grateful to the Reviewer for his/her appreciation of our findings and his/her most positive report. We are hopeful that the constructive suggestions and the proposed corrections improved our manuscript. All the corrections in the revised manuscript are highlighted in yellow for the Reviewer's convenience.

Authors should discuss whether, in addition to phase angle, other parameters (e.g., hand grip strength) can contribute to a more accurate assessment of overtraining

Author’s response: We appreciate Reviewer #2 for the opportunity to address this thought-provoking issue. Indeed, from a multifactorial and interdisciplinary viewpoint, it is not feasible to rely on a single parameter as the sole marker for a complex condition such as Overtraining Syndrome (OTS). On the contrary, the need for more holistic evaluations that integrate a variety of techniques and parameters is increasingly supported in the literature. In this regard, we believe that changes in Phase Angle (PhA) should be interpreted alongside other indicators, such as handgrip strength, to enable a more comprehensive morphofunctional assessment, as has been previously proposed in different clinical settings. While this is not the primary focus of our article, we deemed it important to mention this perspective, as it was suggested.

Authors should explain the meaning and biological significance of the bioimpedance parameters. This reviewer considers that a summary table should be introduced explaining the meaning and biological significance of the bioimpedance parameters.

Author’s response: We thank Reviewer#2 for this important suggestion. A table containing the biological significance of BIA raw parameters and their variations has been included in the revised version (table 1).

Authors should consider including a critical discussion of the limitations of current evidence and explicitly state the gaps in the literature. Highlight the need for targeted longitudinal studies in athletic populations to validate PhA as a reliable marker for overtraining.

Author’s response: We are in complete agreement with Reviewer#2. The limitations of the current literature have been highlighted, and the need for ad hoc studies to confirm our hypothesis has been stressed.

Some typographical errors should be corrected. The meaning of several abbreviations is missing, the authors should correct it.

Author’s response: We thank Reviewer#2. All typos (including the use of abbreviations) have been corrected.

Enhance the reference list with more recent primary studies, especially those focused on the direct assessment of PhA in athletic settings and its relationship with overtraining.

Author’s response: A very recent study demonstrating the relationship between BIA raw parameters (including PhA) with exercise-induced muscle damage has been included.

The term "in lato sensu" could be replaced with "in a broader sense" for better clarity.

Author’s response: We agree with Reviewer#2 with this suggestion, and we changed “in latu sensu” with “in a broader sense”.
